# The synonymous 903C>G mutation in the alpha 1,4-galactosyltransferase gene in a Chinese woman with habitual abortion: A case report: Erratum

**DOI:** 10.1097/MD.0000000000016971

**Published:** 2019-08-16

In the article, “The synonymous 903C>G mutation in the alpha 1,4-galactosyltransferase gene in a Chinese woman with habitual abortion: A case report”,^[1]^ which appears in Volume 98, Issue 31 of *Medicine*, Xiaoying Lv and Dr. Yongquan Chen are co-first authors.
